# Oxidative Stress and Acrosomal Status of Human Spermatozoa Subjected to Hydrophobic Carbon Soot Treatments

**DOI:** 10.3390/nano14050395

**Published:** 2024-02-21

**Authors:** Karekin D. Esmeryan, Ivaylo Rangelov, Todor A. Chaushev

**Affiliations:** 1 Acoustoelectronics Laboratory, Georgi Nadjakov Institute of Solid State Physics, Bulgarian Academy of Sciences, 72, Tzarigradsko Chaussee Blvd., 1784 Sofia, Bulgaria; 2 Specialized Surgical Hospital “Doctor Malinov”, 46, Gotse Delchev Blvd., 1860 Sofia, Bulgaria

**Keywords:** acrosomal status, human spermatozoa, oxidative stress, soot

## Abstract

The fourth industrial revolution extensively reshapes the reality we are living in by blurring the boundaries of physical, digital and biological worlds. A good example is the previously unthinkable incursion of nanoscale waste materials, such as soot, into the technologies for assisted reproduction. Although the rapeseed oil soot may efficiently enhance the progressive motility of human spermatozoa, it is yet unknown whether this material induces undesirable oxidative stress and premature acrosome reaction, endangering the sperm-oocyte fusion and blastocyst formation. In an attempt to clarify this issue, we reveal that the three-hour incubation of human semen mixed with three main types of soot does not cause oxidative stress and spontaneous acrosome reaction of the sperm. These unique findings are attributed to synchronous elimination and stabilization of the oxidants via hydrogen bonding to the acidic groups of the soot (i.e., C=O and/or C-O-C) and electron donation by its basic chemical sites (i.e., C-OH and/or COOH). Moreover, the soot nanoparticles are electrostatically attracted by discrete positively charged areas on the sperm head, increasing its negative charge and in some cases interfering the acrosome reaction. Such novel mechanistic insights emphasize the credibility of rapeseed oil soot to confidently shift from the purely diagnostic and therapeutic phases in reproductive medicine to research dealing with the effect of carbon nanomaterials on the embryo development and implantation.

## 1. Introduction

Reproduction is the key to the existence and continuance of the human race, being intrinsically incorporated into our physiology and the social construct we are living in [1]. Nevertheless, the contemporary social relations and aspirations toward welfare and technological development are inevitably accompanied by mental stress and exhaustion, adversely affecting the male reproductive health and natural conception rates [2]. Therefore, the globally growing male factor infertility is recognized as a huge medical concern, threatening the future survival of the human population and opening new lines of research in reproductive biology [2,3].

Not surprisingly, if regular sexual intercourses within a period of 12 months do not lead to clinical pregnancy, due to impaired spermatogenesis, the infertile couples strongly rely on a positive outcome of assisted reproduction [4,5,6]. This term comprises all clinical methods used to assist in gaining pregnancy, amongst which the classic in-vitro fertilization (IVF) has the highest efficiency, attributed to the opportunity for preliminary selection of both female (oocytes) and male (spermatozoa) gametes [5]. Thus, controlled ovarian hyperstimulation can be implemented to increase the number of oocytes available for IVF, while as an integral part of the assisted reproductive technologies, semen processing via gradient density centrifugation and microfluidic sperm-sorting devices, for instance, facilitates the choice of mature spermatozoa [4,6].

Mature (reproductively active) spermatozoa are those characterized by progressive motility, normal morphology, intact acrosome, DNA integrity and physiological oxidative–reductive balance [7,8,9]. The presence of forward-moving male gametes is a precondition for successful completion of the long travel distance through the female reproductive tract to the oviduct (fallopian tube), in whose widest part (ampulla) the fertilization takes place [10]. During this journey, the “functionally competent” spermatozoon overcomes various obstacles prior to reaching the extracellular matrix of the oocyte, where destabilization of the sperm head occurs [7], inducing rupture of its membrane-bound organelle (acrosome) and leakage of lysine that cleaves the oocyte’s outer wall (Zona Pellucida), as a first step of the sperm–oocyte fusion [8]. Nevertheless, the mature spermatozoa are vulnerable to oxidative stress caused by imbalance between the free radicals (reactive oxygen species (ROS) such as superoxide anion, hydrogen peroxide, hydroxyl radical and singlet oxygen) and antioxidants in the semen, leading to non-specific modification of the unsaturated fatty acids located at the cell membrane [9,11]. Moreover, the high levels of ROS may also inflict sperm DNA fragmentation [9,12], which in turn could delay the blastulation rate (early embryo development) and increase the risk of miscarriage [13,14].

Several procedures are found to be appropriate for alleviating the oxidative stress, DNA damage and decreased sperm motility and, hence, reinforcing the reproductive potential of male gametes. They include an oral intake of antioxidants [15,16,17] and pharmacological phosphodiesterase inhibitors [18,19,20], precise control of the sexual abstinence period [21,22], microfluidic sperm sorting (MSS) [6,23] and varicoceletomy [14,24]. That said, antioxidant therapy helps to offset the DNA fragmentation, but at the expense of reductive stress due to substantial decrease in the oxidation–reduction ratio [14]. Furthermore, phosphodiesterase inhibitors are indeed an excellent tool for preventing the inactivation of intracellular signaling molecules such as cyclic adenosine monophosphate and cyclic guanosine monophosphate, thus enhancing the sperm functionality, but might trigger premature acrosome reaction [19]. In addition, shortening the abstinence interval improves the kinematic parameters of spermatozoa, but decreases their concentration and the volume of seminal fluid [21,22], which could be a problem for patients with low sperm count (oligozoospermia) or reduced sperm motility (asthenozoospermia). Last but not least, while microfluidics and varicoceletomy are provenly effective in diminishing the male factor infertility [23,24], the involvement of expensive equipment and surgical interventions is somewhat inexpedient and painful.

An alternative of the aforementioned methods is a recently invented nanotechnology employing hydrophobic carbon soot nanoparticles, derived by burning rapeseed oil, as a functional activator of human spermatozoa [25,26]. The chemically non-polar soot provides a binary mechanism for enhancing the sperm motility. On one hand, by generating a negatively charged electric field around the gametes and electrostatic repulsive forces that transfer momentum and kinetic energy to the cells, increasing their curvilinear velocity. On the other hand, by altering the enzyme activity in the seminal plasma via the oxygen functional groups of the soot that seem to interact with the enzymes and/or partner molecules, impeding the formation of enzyme–substrate complexes. These physicochemical mechanisms open possibilities for inexpensive and facile adjustment of the functional status of human spermatozoa, but it is still unclear whether this type of soot elicits oxidative stress and/or premature acrosome reaction.

In this study, we advance the state-of-the-art in reproductive biology by analyzing for the first time the oxidative–reduction potential and acrosomal status of human semen preliminary mixed with carbon soot nanoparticles. The novel experimental data revealed in the article ensure new insights regarding the likelihood of boosting the reproductive potential of ejaculated spermatozoa and increasing the success of chosen assisted reproductive technology (e.g., IVF, intrauterine insemination or intracytoplasmic sperm injection—ICSI).

## 2. Materials and Methods

### 2.1. Materials

Zoya Organic&Natural (Sofia, Bulgaria) supplied the rapeseed oil for generating the carbon soot patterns. Microscope glass slides were purchased from Medical Technics Engineering (Sofia, Bulgaria). MIOXSYS analytical device and Human Acrosin ELISA Kit were received from Caerus Biotech (Nyon, Switzerland) and MyBioSource (San Diego, SC, USA) respectively.

### 2.2. Selection Criteria of the Male Subjects

Since the assays were performed in a clinical setting, all patients involved in the experiments were selected primarily on the basis of personal consent without trying to discriminate between those with abnormal and normal sperm parameters. Such an approach allowed us to study the oxidative stress and acrosomal status of a broad spectrum of human semen ranging within normozoospermia, oligozoospermia, asthenozoospermia, oligoasthenozoospermia, etc., which facilitated the gaining of new knowledge regarding the impact of soot on people with deteriorated reproductive health or idiopathic infertility. Informed consents were signed for each ejaculate used in the research, according to the license of Specialized Surgical Hospital “Doctor Malinov” for assisted reproduction activities (ref. № CБ-287/02.11.2021), and the experiments were executed in compliance to the Declaration of Helsinki.

### 2.3. Oxidative–Reduction Potential Assays

The native seminal fluids of four patients were collected in sterile plastic containers following masturbation and after 30 min residence in an incubator, the sperm concentration and motility in the liquefied samples were recorded by dispensing 10 µL seminal droplets in a Makler chamber and scanning three to seven fields via an SCA 6.0.0.2 Mircoptic S. L. software. Then, 200 µg of three types of previously well-characterized soot [25,26], labelled as *soot1-soot2-soot3*, were inserted one at a time in three vials accommodating 200 µL liquefied semen, while a fourth soot-free vial was left as a control. The as-prepared suspensions were incubated for 180 min at ~37 °C, centrifugated at 2000 rpm (relative centrifugal force *RCF* = 268 x*g*, angular velocity *ω* = 209 rad/s) for 5 min and each supernatant (seminal plasma) was removed. Afterwards, the four seminal plasmas and the four sperm aliquots were mixed with SpermWash buffer solution (i.e., overall eight samples) and 30 µL droplets of each sample were placed in the port of disposable platinum-based electrode sensor chips of the MIOXSYS system. The values of oxidative–reduction potential (ORP) were displayed within 2 min and normalized with respect to the sperm concentration [9]. The described procedure was repeated for every patient, warranting the availability of thirty-two values of ORP for further analysis and interpretation. It is important to mention that the buffer did not affect the sensor readings, which was experimentally proven by testing three buffer aliquots containing *soot1*, *soot2* and *soot3*, showing equal ORP within ~220–223 mV. Furthermore, the research was limited to four individuals due to the need for thirty-two chips with a total price of ~$2000, which is the maximum financial resource that can be allocated from a scientific project with a relatively small budget (KP-06-H57/1).

### 2.4. Evaluation of the Acrosomal Status

**Figure 1:**
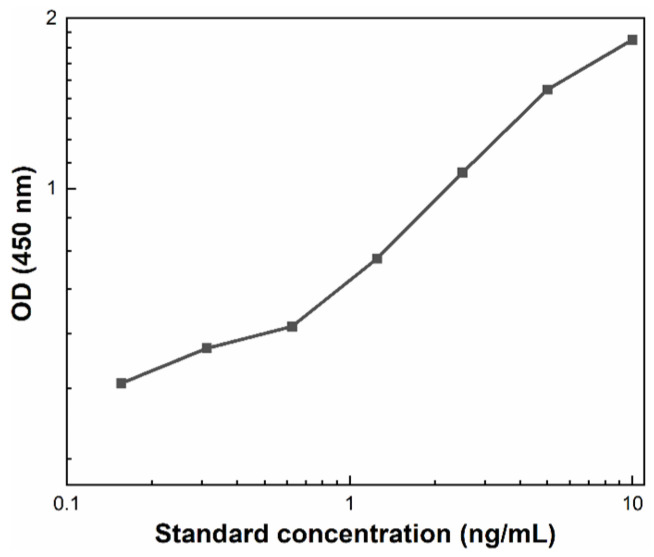
A typical standard curve built by measuring the OD of Standard^TM^ serial dilutions.

These tests were implemented according to the guidelines of MyBioSource Inc. related to the operating principle of Human Acrosin ELISA Kit [27]. Initially, the liquefied semen of ten patients was subjected to computer-assisted sperm analysis and then mixed with carbon soot in exactly the same way described in Section 2.3. Simultaneously, Standard^TM^ solution was reconstituted with 1 mL Sample Diluent^TM^, which produced a stock solution of 10 ng/mL acrosin content. After 15 min equilibration at room temperature, several serial dilutions were made in order to create the so-called standard curve, where 0 ng/mL is the pure Sample Diluent^TM^, while 10 ng/mL is the highest Standard^TM^. Next, 100 µL of Standard^TM^ (all serial dilutions), control (soot-free semen) and soot–semen samples (*soot1*, *soot2* and *soot3*) were placed in a microliter plate, pre-coated with an antibody specific to the target antigen and covered with a plate sealer, and incubated for 120 min at 37 °C. Subsequently, the liquid in each well was removed without washing and 100 µL of Detection Reagent A^TM^ working solution were added in the wells covered again with the sealer. Upon 60 min incubation at 37 °C, the wells were washed three times with 300 µL Wash Buffer^TM^. Later, 100 µL of Detection Reagent B^TM^ working solution were added, and after 60 min incubation at 37 °C, the wells were washed five times. Finally, 90 µL of Substrate Solution^TM^ were inserted in the wells, the plate was incubated for 15 min and 50 µL of Stop Solution (sulfuric acid) were added to terminate the enzyme–substrate reaction. The ensuing color changes were measured spectrophotometrically at a wavelength of 450 nm, while the acrosin concentration (defining the percent of premature acrosomed spermatozoa) was determined by correlating the optical density (OD) of the samples to the standard curve, illustrated in Figure 1, and converting the values via MyCurveFit software.

### 2.5. Statistical Analysis

The statistical dispersion of all experimental data was determined by calculating the mean value and standard deviation of each dataset via Microsoft Excel. The latter was used to run a Single Factor Analysis of Variance (ANOVA) test to compare statistically the means of different data groups, while a post hoc F-test Two Sample for Variances was employed to evaluate the variances between two particular data groups. The derived *p*-values served as a tool for accepting or rejecting the described scientific hypotheses.

## 3. Results

### 3.1. Morphology and Chemistry of the Soot Patterns

**Figure 2:**
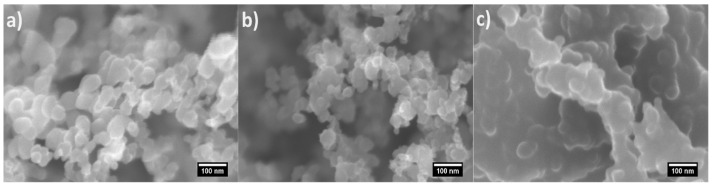
Morphology of (**a**) *soot1*, (**b**) *soot2* and (**c**) *soot3*.

**Figure 3:**
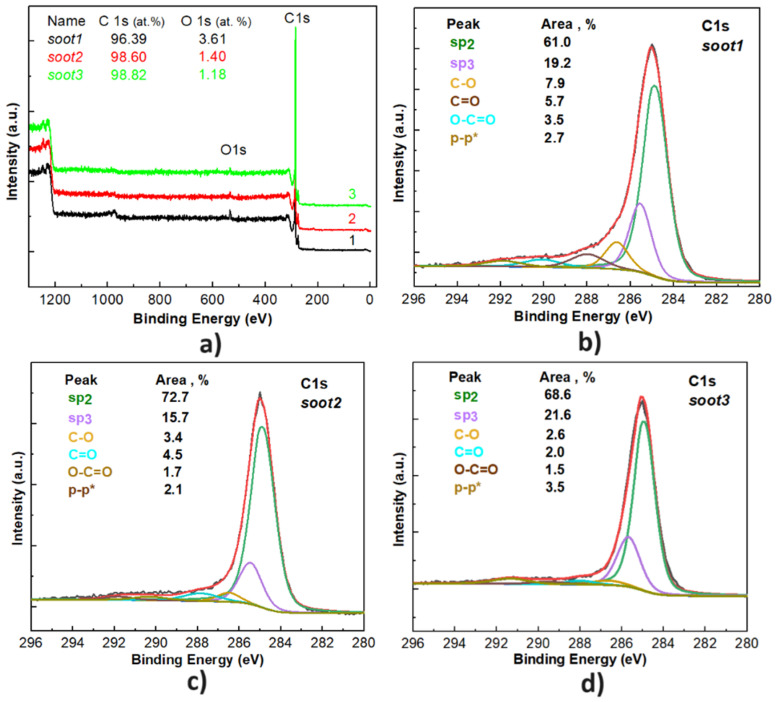
Graphs of X-ray photoelectron spectroscopy (**a**) scan survey and C1s photoelectron core level of (**b**) *soot1*, (**c**) *soot2* and (**d**) *soot3*.

As indicated in Section 2.3, the available soot specimens are fully explored over the last few years of active research (not all relevant literature is cited in the paper to avoid the excessive self-citation) [25,26]. Therefore, we will focus the discussion solely on the main morphological and chemical peculiarities of carbon nanoparticles, introduced in Figure 2 and Figure 3.

What is well-known so far is that the gradual reduction of the incoming air flow rate during the incomplete combustion of rapeseed oil transforms the conventional quasispherical soot (*soot1* in Figure 2a) into semifused nanoparticles (*soot2* in Figure 2b) or enlarged agglomerates (*soot3* in Figure 2c), structured as irregularly shaped fractals. This process coincides with other predictable modifications in the soot, including lowered amount of oxygen functional groups (see Figure 3), enhanced porosity, increased negative zeta potential and sp^3^ bonding [25,26]. Such distinctions do not alter the highly hydrophobic nature of the soot but are expected to influence the oxidative–reductive balance and acrosomal status of the gametes, which is the driving force of our work.

### 3.2. Relationship between the Oxidative–Reduction Potential of Human Semen and Soot Features

**Figure 4:**
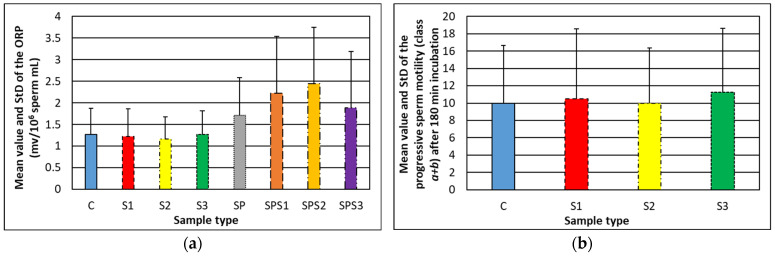
Mean value and standard deviation of the (**a**) ORP of soot-free and soot–semen samples and (**b**) progressive sperm motility after 180 min incubation. Nomenclature: *C*—soot-free spermatozoa (first control); *S1*—spermatozoa mixed with *soot1*; *S2*—spermatozoa mixed with *soot2*; *S3*—spermatozoa mixed with *soot3*; *SP*—soot-free seminal plasma (second control); *SPS1*—seminal plasma mixed with *soot1*; *SPS2*—seminal plasma mixed with *soot2*; *SPS3*—seminal plasma mixed with *soot3*.

Perfectly balancing among the production of oxygen molecules and their timely removal from the semen mixed with rapeseed oil soot is the key to terminate the inordinate release of superoxides that transform into free radicals inflicting damage to the mitochondria and other cellular structures [28]. Figure 4 shows how the soot nanomaterials affect the total oxidant–reductant activity in the sperm and seminal plasma of four patients.

It can be seen that the ORP of male gametes treated with soot does not differ substantially from that of the control specimens (see Figure 4a) and most importantly, all values are below the cut-off range (1.38–1.41 mv/10^6^ sperm mL), categorizing the patients as infertile with abnormal seminal characteristics [9]. Moreover, impaired oxidant–reductant activity is noticed in the seminal plasmas incubated with *soot1–soot2–soot3* compared to the second control (see Figure 4a), hinting at the occurrence of some interesting from scientific point-of-view chemical interactions in the ejaculates containing rapeseed oil soot. Expectedly, the incubation period of 180 min decreases the progressive sperm motility (see Figure 4b), which decreases to ~10 ± 1% due to the depletion of nutrients in seminal plasma [25]. 

### 3.3. Effect of Rapeseed Oil Soot on the Acrosome Reaction of Human Spermatozoa

**Figure 5:**
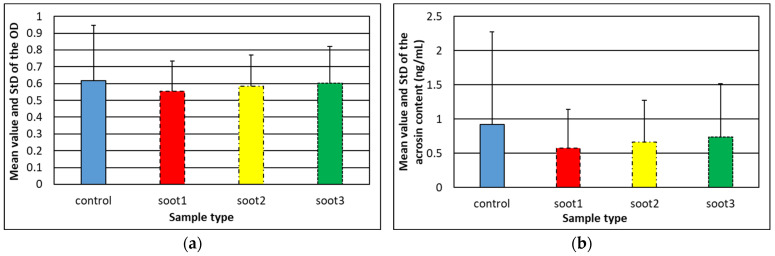
Mean value and standard deviation (StD) of the (**a**) optical density and (**b**) acrosin content of soot-free (control) and soot-treated semen after Human Acrosin ELISA Kit assays. Both figures reveal similar tendencies, but the difference between control and soot-treated samples in Figure 5b is more pronounced due to a cumulative effect, since the OD-acrosin dependence is logarithmic (see Figure 1). The large StD in control specimens reflects the inherent diversity of native human ejaculates of all patients involved in the study (see Section 2.2).

The acrosome reaction is an exocytotic event leading to eversion of the acrosomal vesicle and secretion of hydrolytic enzymes, creating “a tunnel” in the oocyte’s Zona Pellucida, through which the spermatozoon reaches and reshapes the oocyte’s plasma membrane [8,29]. Depending on the factors triggering acrosome reaction, it can happen spontaneously via a rise in the intra-acrosomal pH and Ca^2+^ ions content or being enabled by natural inductors such as follicular fluids, progesterone and neurotransmitters. Therefore, in this section we aim to elucidate whether the rapeseed oil soot can or cannot act as an inhibitor of the spontaneous acrosome reaction, which is crucial for the future applicability of this nanomaterial to reproductive medicine. It should be mentioned that induced acrosome reaction events are not considered herein, because the experimental setup described in Section 2.4 excludes the contribution of follicular fluids, progesterone or neurotransmitters. Figure 5 summarizes the first documented attempts of analyzing the acrosomal status of human spermatozoa after hydrophobic soot treatments.

Obviously, the obtained experimental results are exciting and unforeseen, because the average OD and acrosin content in the ejaculates incubated with soot is lower (as an absolute value) compared to the controls, implying that these carbonaceous nanoparticles may have the ability to suppress the spontaneous acrosome reaction—a hypothesis elaborated in the next section. Furthermore, the probability for statistical difference among the individual groups in Figure 4 and Figure 5, provided by the Single Factor ANOVA test, is *p* = 0.58–0.86 (see the document entitled “*ORP & Acrosome_statistical analysis*” in Supplementary File S1), indisputably confirming that the rapeseed oil soot patterns do not adversely affect both the oxidative–reductive balance and acrosomal status of the male gametes, making this material reliable enough for use in reproductive biology. Moreover, the outcome of F-test Two Sample for Variances indicates statistically significant deviations between some numerical values in the data groups *C-S1*, *C-S2* and *C-S3* in Figure 5 (*p* = 0.008–0.05; see the document entitled “*ORP & Acrosome_statistical analysis*” in Supplementary File S1). For instance, the acrosin content in the ejaculates of *Patients 1, 5* and *6* decreases by a factor of 1.6–2.3 after the insertion of *soot1* nanoparticles, which means that in nearly 30% of cases, this type of soot impedes the spontaneous acrosome reaction. As a result, preserving the acrosome intact prevents the loss of acrosomal enzyme activity during the bursting in the female genital tract of the cap-like structure covering the anterior portion of the sperm nucleus, thus increasing the chance for successful in-vitro fertilization [30]. 

## 4. Discussion

The primary mechanism of nanotoxicity of soot aerosols considers the hydrophilicity of soot nanoparticles and the abundance of oxygen sites on their surface, serving as points of contact with the hydrophilic biomolecules of living cells [31]. It is known that human spermatozoa are highly vulnerable to oxidative stress, as they have insufficient antioxidant defense and DNA repair capabilities, enabling ROS formation by the seminal leukocytes and residual cytoplasm around the sperm midpiece [9,32].

Based on the results from surface characterization, the rapeseed oil soot employed in this research is composed of various oxygen functional groups (see Figure 3) such as epoxy/hydroxyl (C-O-C/C-OH) and carbonyl/carboxyl (C=O/COOH), divided to electron donors (Lewis bases, also known as nucleophiles) and electron acceptors (Lewis acids, also known as electrophiles) [33]. Knowing that the soot interacts with oxygen-containing molecules (e.g., liquid water, water vapor, etc.) primarily via hydrogen bonding [33], the presence of hydroxyl radicals (*OH) in spermatozoa promotes free electron pair donation by these hydroxide anions, creating hydrogen bonds with the carbonyl (C=O) and epoxy (C-O-C) complexes of the soot. In our particular case, the weak chemical polarity of the rapeseed oil soot, due to its very low surface oxidation (O ≤ 4 at.%, according to Figure 3), hinders the excessive accumulation of free radicals in the sperm, but in the meantime contributes to the chemical neutralization of ROS by the present oxygen functionalities. In turn, the oxidative–reductive balance in spermatozoa remains unchanged and the harmful oxidants are accumulated mostly in the seminal plasma. This statement is correct, because the average values of ORP in the seminal plasma are twice as high compared to those in the male gametes and when the soot oxidation increases, the ORP of spermatozoa slightly decreases—especially true if comparing *soot1* and *soot3* specimens (see Figure 4a). Additionally, the stabilization of ROS requires electron donation from proteins, lipids and DNA molecules [28], so the carboxyl (COOH) and hydroxyl (C-OH) groups of the rapeseed oil soot (see Figure 3) could act as an antioxidant by donating an electron pair and thus eliminating the ROS from the biological system [32].

To outline the pathways of soot-mediated blocking of the acrosome reaction, we will discuss a few important biochemical and physiological alterations that the spermatozoa experience in their travel throughout the female reproductive tract [34,35]. When the gametes are away from the egg, they are highly negatively charged and the capacitation and acrosome reaction are impeded by the cholesterol and Zn^2+^ ions in the seminal plasma [35] or via molecules of sialoglycoproteins, sialic acid, steroid sulfates and sulfated carbohydrates attached to the sperm body [34]. When approaching the oocyte, the spermatozoa undergo maturation, associated with increased permeability of the plasma membrane to calcium, stimulating the acrosome reaction. The next steps involve reduction of the sperm negative charge to pass the cumulus–oocyte complex and appearance of discrete positively charged areas on the head, allowing the sperm–egg fusion—a membrane potential-mediated process, at which the fertilized oocyte rapidly shifts its potential from negative to positive and shields electrically the polyspermy [34].

From that point-of-view, the net charge of reproductively mature spermatozoon is a dynamic feature regulating its fertilizing ability, depending on the developmental phase. Although the male gametes are negatively charged, recent findings reveal that their surface accommodates discrete positively charged regions attracting electrostatically SiO_2_ nanoparticles with negative zeta potential [34]. Specifically, the SiO_2_ particles bind preferentially to the plasma membrane and sperm head (where the acrosome is located) and rarely to the flagellum or entire cell’s surface, in contrast to the positively charged Fe_2_O_3_ particles.

Since the rapeseed oil soot is negatively charged and incorporates oxygen (an atom with high electronegativity) [25], the oxygen functionalities of soot nanoparticles interact electrostatically with the sperm cells possessing intact acrosome, stabilize their membrane, increase the negative charge and inhibit the acrosome reaction, identically to the sulfate-based compounds [34]. Such a hypothesis is supported by the fact that the gradual decrease in the soot oxidation leads to weaker suppression of the acrosome reaction (see Figure 3 and Figure 5b). Moreover, the described mechanism has scientific meaning, because the capacitation proceeds with removal of cholesterol from the phospholipid bilayer and loss of binding affinity to the negatively charged particles [34]. In other words, the sperm already being at the fertilizing stage (capacitated and acrosome reacted) is not electrically attracted by the soot.

Another explanation of the experimental results is related to the decreased enzyme activity in the seminal plasma and/or spermatozoa, governed by the soot [26]. In particular, the capacitation of mammalian sperm in-vitro necessitates glycolysis (extraction of energy from glucose by dividing it into pyruvates) to supply dynein adenosine triphosphate (ATP) and lactate dehydrogenase (LDH) enzymes catalyzing the reversible interconversion of pyruvate and lactate [36]. Thus, weakening the LDH activity [26] is an alternative pathway for blocking the capacitation and acrosome reaction.

## 5. Future Perspectives

Objectively speaking, the zeta potential, surface oxidation and particle size of rapeseed oil soot patterns change in a traceable manner the functional competence of human spermatozoa. However, while the motility and enzyme activity are greatly affected by the least oxidized soot (i.e., *soot3*) [25,26], the oxidative–reductive balance and acrosomal status of the sperm is maintained much better by the highly oxidized quasispherical carbon nanostructures (i.e., *soot1*). Such contrasting outcomes outline the need of performing more experiments with an aim to elucidate entirely the underlying functional mechanism of soot. The future research should encompass parallel determination of all sperm parameters after incubation with soot, including motility, full biochemistry, ORP, acrosomal status, morphology and DNA fragmentation, since this will help to understand the interactions regulating the fertilizing ability of human semen.

## 6. Conclusions

Incubating human seminal fluids with hydrophobic carbon soot nanomaterials, differing by morphology, chemical state and zeta potential, resulted in unaltered oxidative–reduction potential and relatively equal percentage of acrosome-reacted spermatozoa compared to the controls. These surprising observations were attributed to the degree of soot oxidation, governing the neutralization of oxidants produced by the male gametes via hydrogen bonds between the hydroxyl radicals and carbonyl/carboxyl functional groups of the soot and via donation of an electron pair to the free radicals. Quite beneficial in this regard was found to be the highly oxidized soot possessing quasispherical particle morphology (i.e., *soot1*), preserving also to the highest extent the acrosomeintactness. Such negatively charged soot created an electrostatic field around the cells, favored the attraction of nanoparticles to isolated positively charged areas on the sperm body and stabilized the plasma membrane. The new knowledge disclosed herein provides a rational strategy enriching the existing postejaculatory sperm treatment approaches for the purposes of assisted reproduction.

## Data Availability

All data needed to evaluate the conclusions in this paper are presented duly. Additional data related to this research may be requested from the authors.

## Supplementary Materials

The following supporting information can be downloaded at: https://www.mdpi.com/article/10.3390/nano14050395/s1, File S1: An Excel document entitled “*ORP & Acrosome_statistical analysis*”, incorporating overall statistical analysis, including the Single Factor ANOVA tests and F-Tests Two Sample for Variances, of both the oxidative–reduction potential and acrosome reaction assays.
